# Higher Medical Education

**Published:** 1892-08

**Authors:** 


### HIGHER MEDICAL EDUCATION.

To all those who are interested in medical education any improvement in the facilities for teaching the science and art of medicine can but furnish a gratifying spectacle. We therefore note with pleasure the efforts on the part of the medical colleges of our city to increase the advantages which they have to offer to their students. The erection of an entirely new building by one college and the fitting up of chemical and microscopical laboratories by the other are indications of a progressive spirit which seeks to keep abreast of the great advances which are constantly taking place in the medical art. The day is past when the study of medicine consisted in sitting for six or eight hours a day on hard benches and listening to didactic lectures. The eye and the hand now participate in the acquirement of knowledge as well as the ear.

But in the rapid progress of medical instruction we are liable to lose sight of the fact that the training of the eye, the ear and the hand are but the means to an end. It is not enough that the student be taught to detect a crepitant *râle*, to make a quantitative estimate of the amount of sugar in the urine or to distinguish between a streptococcus and a gonococcus under the microscope, unless he be taught at the same time by a logical process of reasoning to deduce from the premises furnished by the senses a conclusion which shall constitute a diagnosis and form the basis for a rational method of treatment. The ability to conduct such a process of reasoning presupposes on the part of the student an amount of mental training equal and very similar to that involved in the solution of a mathematical problem. Such training is the result of long and systematic academical study. Without such training the acquirement of a knowledge of the science of medicine is as impossible as would be the mastery of the calculus to a man who did not know the multiplication table. In the lack of such preliminary training lies the explanation of the fact that men are sent out every year from our medical colleges whose whole stock of knowledge consists of a list of diseases each labeled with an appropriate remedy. The diplomas issued to such men are falsehoods and the Latin in which the false statements are concealed does not lessen their enormity.

But grant that a man has a good preliminary mental equipment when he enters upon the study of medicine, what lies before him to be accomplished? Intelligent students often say that during their first year of study they are appalled as they see opening out before them the vista of knowledge yet to be acquired. In the ten months of attendance upon lectures required by our colleges, what mind, be it ever so brilliant, can make more than a beginning. If we compare the number of branches taught, their complexity and difficulty with a year's curriculum in any literary college, the absurdity of the whole plan is at once apparent. And yet our colleges assume to believe that a man who is fresh from the plough or the mechanic's bench and whose mind has never been trained to think closely or correctly, will in ten months of study so thoroughly master seven difficult branches of knowledge as to be worthy to be called a *doctor*—a teacher—of those branches.

What is the remedy for those evils? The remedy has already been discovered and applied in many of the medical colleges in this country. It lies in a preliminary examination as to the applicant's fitness to pursue the study of medicine and a lengthened course of study as a requirement for graduation. The beneficial effects of such a change of plan are most markedly apparent in the class of practitioners sent out by colleges which have taken this step. Their graduates are an honor to their *alma mater*

and to the profession. They are in no danger of bringing the practice of medicine into disrepute by their ignorance and incompetency, but on the contrary, they help to elevate it to its proper plane as one of the learned professions.

We trust that the Atlanta colleges will soon see fit to take this grand step forward. With a good hospital for clinical teaching, with improved methods and facilities for instruction, and with competent and earnest teachers, all that is now needed to give to Atlanta the prominence which she merits as a medical center, is that our schools shall require a three or four years' course of study and that none but educated men shall be accepted as students.

